# A method to enhance privacy preservation in cloud storage through a three-layer scheme for computational intelligence in fog computing

**DOI:** 10.1016/j.mex.2024.103053

**Published:** 2024-11-19

**Authors:** Sneha Ojha, Priyanka Paygude, Amol Dhumane, Snehal Rathi, Vijaykumar Bidve, Ajay Kumar, Prakash Devale

**Affiliations:** a Bharati Vidyapeeth (Deemed to be University), College of Engineering, Pune, India; b Symbiosis Institute of Technology, Symbiosis International University, Lavale, Pune, India; c BRACT'S Vishwakarma Institute of Information Technology, Pune, India; d School of CSIT, Symbiosis Skills and Professional University, Kiwale, Pune, India; e Manipal University, Jaipur, India

**Keywords:** Cloud computing, Fog computing, Privacy preservation techniques, Encryption, Hash solomon code, Hash Solomon Algorithm based on Reed Solomon Algorithm

## Abstract

Recent advancements in cloud computing have heightened concerns about data control and privacy due to vulnerabilities in traditional encryption methods, which may not withstand internal attacks from cloud servers. To overcome these issues about the data privacy and control of transfer on cloud, a novel three-tier storage model incorporating fog computing method has been proposed. This framework leverages the advantages of cloud storage while enhancing data privacy. The approach uses the Hash-Solomon code algorithm to partition data into distinct segments, distributing a portion of it across local machines and fog servers, in addition to cloud storage. This distribution not only increases data privacy but also optimises storage efficiency. Computational intelligence plays a crucial role by calculating the optimal data distribution across cloud, fog, and local servers, ensuring balanced and secure data storage.

• Experimental analysis of this mathematical mode has demonstrated a significant improvement in storage efficiency, with increases ranging from 30 % to 40 % as the volume of data blocks grows.
• This innovative framework based on Hash Solomon code method effectively addresses privacy concerns while maintaining the benefits of cloud computing, offering a robust solution for secure and efficient data management.

## Graphical abstract

**Figure:**
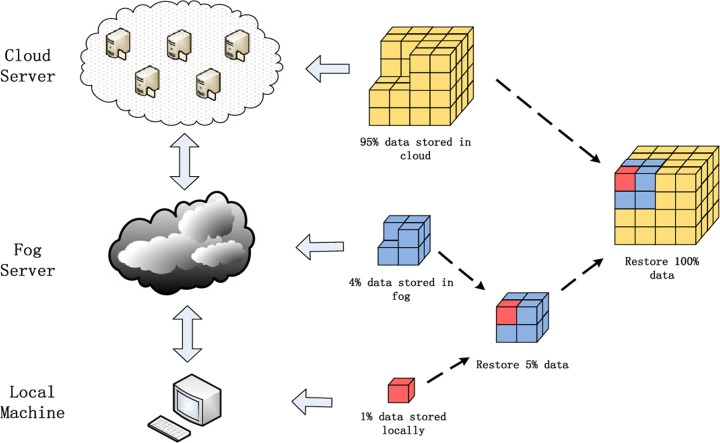

Specifications table

**Table:** 

Subject area:	Computer Science
More specific subject area:	*Cloud Computing, Security and Privacy of Data*
Name of your method:	*Hash Solomon Algorithm based on Reed Solomon Algorithm]*
Name and reference of original method:	*Reed Solomon Method]*
Resource availability:	*Not Available].*

## Background

The motivation for this research stems from the escalating concerns over data privacy and security in cloud computing. Traditional encryption methods are increasingly vulnerable to internal attacks from cloud servers, posing significant risks to data control and privacy. With the rise in cyber threats and stringent data protection regulations like GDPR and CCPA, there is an urgent need for advanced data protection mechanisms. Furthermore, traditional cloud computing creates a single point of vulnerability and control issues, where users have limited oversight over their stored data, raising fears of unauthorized access and misuse by cloud providers. To address these challenges, the research proposes a novel three-tier storage model that incorporates fog computing, leveraging its edge processing capabilities to enhance data security and reduce latency. By using the Hash-Solomon code algorithm, data is partitioned into distinct segments and distributed across local machines, fog servers, and cloud storage. This multi-layered approach not only heightens data privacy by making it more challenging for attackers to access the entire dataset but also optimizes storage efficiency. The integration of computational intelligence further strengthens the system by dynamically calculating the optimal distribution of data across the different storage layers, ensuring balanced and secure data storage that adapts to varying conditions. Empirical evidence supports the effectiveness of this model, demonstrating a significant 30 % to 40 % improvement in storage efficiency as data volumes grow. This innovative framework offers a robust solution to the privacy concerns inherent in cloud computing while retaining its advantages, providing a scalable and secure method for efficient data management.

## Method details

### Introduction

In the field of computer science, cloud computing refers to the practice of outsourcing computer services, such as power. Users are only required to exploit it. They consider power generation and transmission to be insignificant. Monthly bills encompass their consumption. Cloud computing enables users to leverage data storage, computational capabilities, and specialized application development environments without incurring the need for comprehensive knowledge of the underlying infrastructure [1]. The prevalence of online cloud computing is widespread. Within computer network designs, the term “the cloud” is used to symbolize the Internet, concealing its intricate structure. This computing approach enables clients to conveniently access technology-enabled services through online platforms, sometimes referred to as “in the cloud,” without the need for server or infrastructure management. Fog computing is a phenomenon that arises from the increasing challenges associated with achieving unbiased data access in expansive cloud systems and big data platforms [2]. The acquisition of data of substandard quality is observed.

The influence of fog computing on cloud and big data platforms is yet uncertain. Various precision metrics have been devised to address the challenges associated with accurately transmitting material. The functionality of fog networking is dependent on the control plane and data plane. Fog computing relocates data-plane computing services in closer proximity to the network edge, as opposed to being hosted on servers within a data center [1]. Fog computing places significant focus on proximity to end users and client objectives, a dense geographical dispersion, and the consolidation of resources in local areas, as well as the implementation of redundancy measures to mitigate any failures. Edge analytics and stream mining have the potential to enhance customer Quality of Service (QoS), minimize latency, and conserve bandwidth on the front end.

The CSP is responsible for managing the data rather than the user. Therefore, individuals are unable to ascertain the specific location of their data storage, potentially impacting the ownership and continuity of data management [1]. The CSP has unrestricted access to and can search cloud-based data. In the event of a breach in the CSP server, malicious actors can get user data. Both options pose the danger of compromising or misplacing crucial data. The majority of cloud data security approaches impose limitations on user access or employ encryption for all stored data. These solutions can effectively address the majority of these difficulties. Although there have been advancements in algorithms, none of these strategies are capable of preventing an internal attack. In this paper, we proposed a three-layer security approach using Hash Solomon code which was inspired by Reed-Solomon code [2,3].

Fog computing employs several fog nodes to enhance the capabilities of cloud computing. These nodes can store and process data. The user data is safeguarded by distributing it among the fog server, cloud server, and local PC in our system. The Hash-Solomon method ensures that partial data is incapable of reconstructing actual data. Nevertheless, Hash-Solomon encoding generates redundancy that can be deliberately taken advantage of during the decoding process. The use of redundant blocks inside a storage system enhances reliability but necessitates increased storage space for data. Our methodology ensures the safeguarding of users' data through the fair distribution of data. CI can streamline the complex computations involved in the Hash-Solomon method. Recently, CI models (WSNs) have successfully addressed a range of issues, such as wireless sensor networks [4]. CI offers intelligent adaptive methods in dynamic environments such as WSNs [5]. We utilize CI for fog mathematics in our work. The efficacy of our internal privacy solution surpasses that of existing approaches, particularly for CSPs [6].

The remaining sections of this paper is structured as follows: Section 2 provides a comprehensive assessment of the existing literature, Section 3 offers a detailed explanation of the suggested approaches, and Section 4 presents a detailed description of the modules. Section 5 focuses on the experimental setup, whereas section 6 presents the experimental analysis and results. The paper is concluded in Section 7.

## Literature review

This paper focuses on 3 tier storage using fog layer to overcome the issues for privacy preservation in cloud computing. The main aim is to protect the user's information while using the cloud computing storage of data. Reed Solomon code based on Hash Solomon was used in the proposed framework to segment the data in three layers. From this segmented data, some data was put on local machine and some on fog service layers. With the help of the computational intelligence algorithms, the system decided the optimal way of allocating data to all 3 layers which includes cloud, local machine and fog layers. The proposed mechanism which was earlier in theory only, was tested and implemented with practical scenario for security and privacy purpose, & found it beneficial to render the applicability of the system [[6], [7], [8], [9]].

In the present system, the Reed-Solomon code-based encoding concept was utilized for the creation of a matrix, thus making it possible to divide the data of encrypted file into different segments. The segmented data was allocated in fog, cloud and local machine layers, primarily and majorly located on cloud server. The proposed system lowers the burden of storing data on local machine while storing maximum data on cloud server [10].

Poojita et al. presents a novel storage system that combines fog computing and cloud computing for storing data with enhanced privacy protection. The segmentation of data was done with the Hash-Solomon code algorithm where data was distributed in 3 parts. 95 % was stored on cloud, 4 % on fog and only 1 % in local machine as secondary storage or main memory. Hash Solomon code further enhances the privacy and security of data-by-data encryption before and after the segmentation. Privacy protection is further bolstered by the Hash-Solomon algorithm, which ensures data encryption both pre- and post-segmentation. Cauchy or Vandermonde matrix was utilized for encoding the matrix for the data encoding performance [11].

Ahsan et al. presented a cloud and fog based online storage system to protect the data from anonymous users. X-OR encryption combination was used to hide the data and further block management approach was used to avoid unusual activities of retrieving the data [12].

The utilization of randomly generated files for experimental purposes demonstrates the resilience and efficacy of the suggested methodology. In general, the research introduces a privacy-preserving model that incorporates fog computing to better the security of cloud data storage. This model demonstrates enhanced encryption and decryption performance in comparison to prior methodologies [13].

Kamble et al. in their work presented a data outsourcing strategy designed for (IoT) devices within the context of fog computing. The scheme aims to provide both privacy protection and computation verification. Current solutions primarily concentrate on performing operations on encrypted data, but they cannot verify computations. The strategy being offered is founded upon an encrypted database system that possesses linear computing capabilities and exhibits efficient query ability. The approach incorporates homomorphic message authenticators into the interlayer programme to facilitate computational verification. The technique enables the system to verify whether the cloud delivers the desired service accurately. The experimental results demonstrate that the suggested approach exhibits comparable effectiveness to the original design while incurring minimal additional time stress [14].

The research presented by Gai et al., expands upon a previous fog-based framework and presents significant distinctions in the FAF model. The research funded by the B.I.T.Research Fund Programme for Young Scholars provides funding for this study [15].

In today's scenario, one has understood the importance and significance of Cloud computing. Although it is beneficial and low-cost storage technique, it faces issues of privacy protection. One has to depend on encryption technology to protect the data from internal and external attacks [16].

To overcome on this issue, the research presented in this study uses the fog computing approach using the Hash-Solomon coding algorithm. The proposed approach stores a very small amount of data in local machines and major section on fog and cloud layers. The suggested scheme's practicality is confirmed by theoretical safety analysis and experimentation, establishing it as a robust addition to current cloud storage techniques [17].

## Proposed methodologies

Fog-based computing with Three Layer Storage (TLS) [12] to ensure user privacy was proposed by the author in this section. The TLS framework offers consumers a degree of autonomy and safeguards their privacy. As previously said, internal attacks are challenging to withstand. Traditional methods are effective in countering exterior dangers, but they are ineffective against CSP. In contrast to conventional methodologies, our approach employs a method of encoding user data into three distinct segments of varying widths. Secrecy will cause each individual to disregard crucial information.

When the integration of cloud computing and fog computing occurs, the data is stored in a descending sequence of size by the fog server, cloud server, and the user's local workstation. This method effectively hinders an assailant from recreating user data, even if they possess complete access to all server data. The CSP is unable to collect pertinent data in the absence of access to the user's workstation and fog server.

**Fig. 1:**
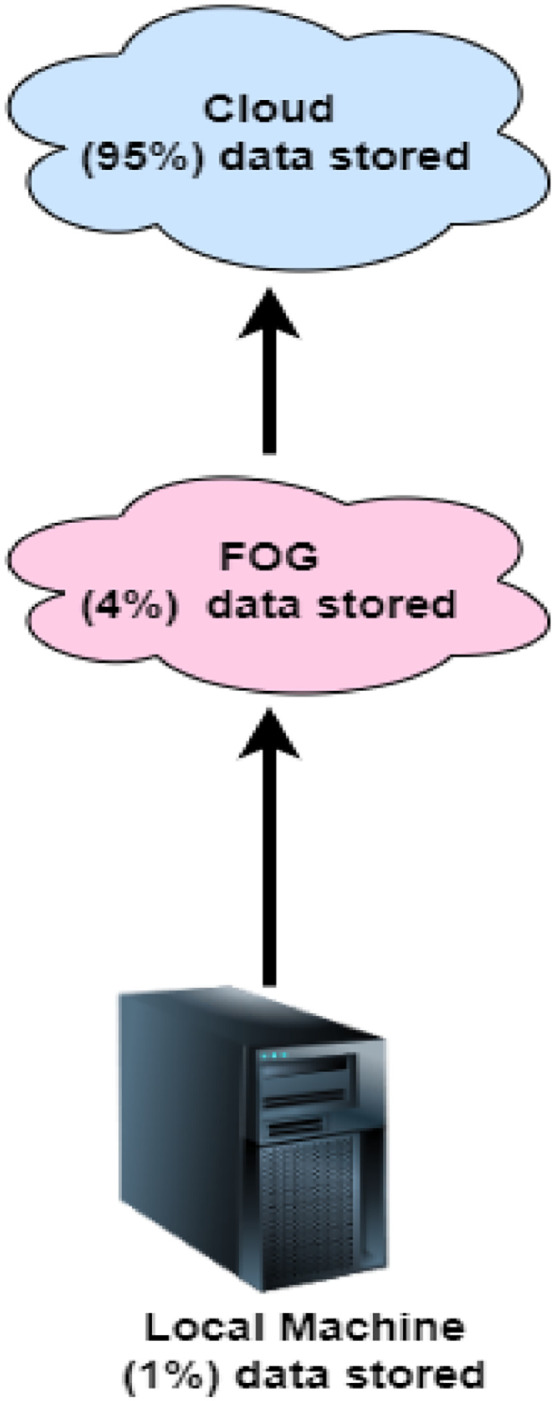
Three Layer System Architecture based on Fog Layer.

Fig. 1 displays the three-layer system architecture based on the fog layer. The utilization of (TLS) is recommended to augment the storage and processing capabilities of the fog server. The system consists of the fog server, cloud server, and local PC. The allocation strategies employed by users have an impact on server storage. The initial step is to encrypt user data stored on their device. To illustrate the notion, the computer may store data with a margin of error of 1 %. The fog server should receive 99 per cent of the data. The fog server employs a consistent approach to processing machine data presented by the user. Following the retention of a restricted quantity of data on the fog server, the remaining data will be sent to the cloud. Hash-Solomon coding is employed in all of the methods. The Hash-Solomon code functions as a variation of the Reed-Solomon code [13,15]. The Hash-Solomon encoding algorithm partitions the data into k subsets and produces m additional subsets.The unique feature of the Hash-Solomon code allows anyone given k out of m bits of information to reassemble the complete set. Therefore, it is not possible to replicate the full dataset using less than k data points. Our technique restricts the storage capacity of the top server to a maximum of one piece of data. The final server contains the remaining data. Despite obtaining data from any of the three levels, a thief is unable to fully reconstruct the image. This ensures the security of user data.

### Hash- Solomon Code algorithm

The Hash-Solomon method, known for its efficiency, enhances the storage and retrieval of data while simultaneously safeguarding anonymity. This technology guarantees data secrecy by dividing it into various portions and dispersing it over multiple websites. In order to execute the method, the data should be divided into k segments. The Reed-Solomon technique, which corrects errors, is used to encode and recover inaccurate data in discrete segments. The quantity of storage locations for encoded data depends on the production of duplicate components. Assuming that k is equivalent to 3 and m is equivalent to 2, the data will be evenly allocated among 5 sites. The Reed-Solomon method, proposed by Kadir W. et al. in 2023, utilizes additional data to restore missing or damaged data components. If the recovery attempt is unsuccessful, the algorithm will then proceed to reconstruct the data by utilizing the remaining available data. The hash-Solomon coding scheme is utilized to ensure data confidentiality by fragmenting and storing data in separate places. The use of many storage locations improves the efficiency of storing and retrieving data [18]. Furthermore, this system is easy to implement. Nevertheless, it is crucial to consider the constraints of the Hash-Solomon coding scheme. The computational requirements for Reed-Solomon encoding and decoding are significant. Superior data privacy protection methods exist in comparison to the algorithm under consideration. The Hash-Solomon coding technique is a very efficient method for ensuring data privacy and improving the efficiency of storage and retrieval procedures.

The main purpose of the Hash-Solomon coding algorithm is to divide data into separate parts [11,19]. The data is saved in three locations. Information is classified according to its importance in order to enhance understanding.

Three-layer privacy preservation using hash Solomon code (HSC) generally aims to enhance security and privacy in data storage and transmission systems. This method combines cryptographic hashing, Reed-Solomon codes, and other techniques to ensure data integrity, confidentiality, and availability. Here's a detailed explanation of how security is achieved through these three layers.

In Layer 1 Data is processed through a cryptographic hash function (e.g., SHA-256), which produces a fixed-size hash value (digest) unique to the data [20]. From security perspective, it is computationally infeasible to generate the same hash value from two different inputs (collision resistance). Any change in the input data results in a significantly different hash value (avalanche effect). Layer 2 is Reed Solomon Code based HSC where data is divided into blocks and encoded using Reed-Solomon codes. These codes add redundancy by generating additional parity blocks. Reed-Solomon codes can detect and correct multiple errors within the data blocks, providing robustness against data corruption and ensuring data availability even if parts of the data are lost or corrupted. Layer 3 is an encryption algorithm. The data (or the data combined with parity blocks from Reed-Solomon encoding) is encrypted using a symmetric (e.g., AES) or asymmetric (e.g., RSA) encryption algorithm. Encryption ensures that only authorized parties with the correct decryption key can access the data. It protects against unauthorized access and eavesdropping.

**Fig. 2:**
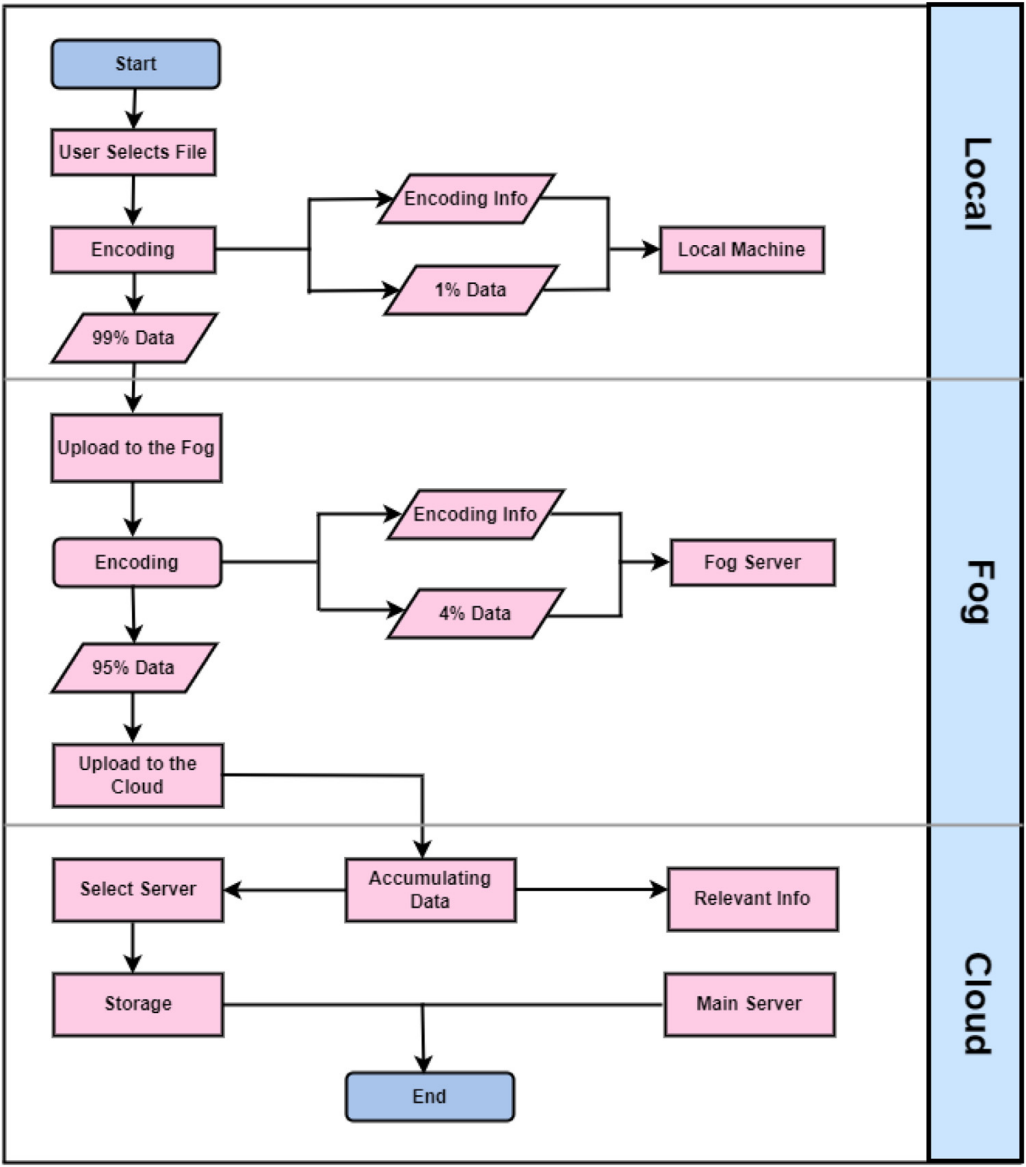
The Storage Process.

The storage process in the flowchart (Fig. 2) involves multiple stages across local, fog, and cloud environments. Initially, the user selects a file and performs encoding on their local machine. This encoded data is split, with 1 % of the encoding information stored locally and 99 % of the data being prepared for upload to the fog layer. Once the data reaches the fog server, it undergoes another round of encoding. Here, 4 % of the encoding information is retained at the fog level, and the remaining 95 % of the data is sent to the cloud for further processing. In the cloud, the data is accumulated, analyzed, and relevant information is extracted. The relevant information is directed to the main server, while the accumulated data undergoes a selection process to determine the appropriate server for storage. Finally, the selected server stores the data, completing the process. This multi-tiered approach ensures efficient data management and storage across different layers, optimizing the use of local, fog, and cloud resources.

The uniqueness of the three-layer approach for computational intelligence in fog computing resides in its ability to enhance privacy preservation in cloud storage by integrating advanced strategies specifically designed to tackle the distinct issues presented by fog computing environments.

1. **Layered Approach:** Incorporating a three-layer scheme, which generally includes cryptographic hashing, error correction codes such as Reed-Solomon, and encryption, establishes a strong foundation for data security. This approach ensures data integrity and availability. This layered framework effectively safeguards information against various threats.
2. **Decentralized Environment:** Fog computing brings cloud services closer to the network edge, presenting distinct challenges like heterogeneous devices, diverse network conditions, and limited computational resources. This extension to the edge introduces complexity in managing these varied environments. Consequently, it requires addressing these unique issues for effective implementation.
3. **Local Processing:** Utilizing local processing, the scheme minimizes latency and bandwidth usage, essential for time-sensitive fog computing applications. This local approach enhances efficiency and responsiveness. As a result, it is crucial for optimizing performance in such environments.

## Module description

### Registration module

Users can register their login ID with this module by providing only the essential personal information. Users express a preference for being requested to provide only essential information, such as their email address, name, mobile phone number, and password. To facilitate their access to the website.

### Login module

Within this specific segment, individuals have the opportunity to enter their login credentials and password in order to gain access to the website. It limits access to just authorized users. The website is protected by a password and can only be viewed by people who have been verified and validated.

### Storage module

This component provides customers with the choice to employ one of three cloud storage services for data backup. Data can be kept in several locations, such as cloud-based systems, fog computing, or local servers. The majority of our data, specifically 80 %, is stored on a cloud server. The sensitivity level of the data stored in the fog server is limited to 15 %. Only 5 % of the available information is stored on the local server.

### Recovery module

Users can choose to recover lost data from one of three servers that are currently accessible. The BCH algorithm will include the data in the bucket if it meets these three conditions. Individuals can efficiently access their data from the bucket framework, even in the event of a breach occurring at any of its layers.

### Download procedure

**Fig. 3:**
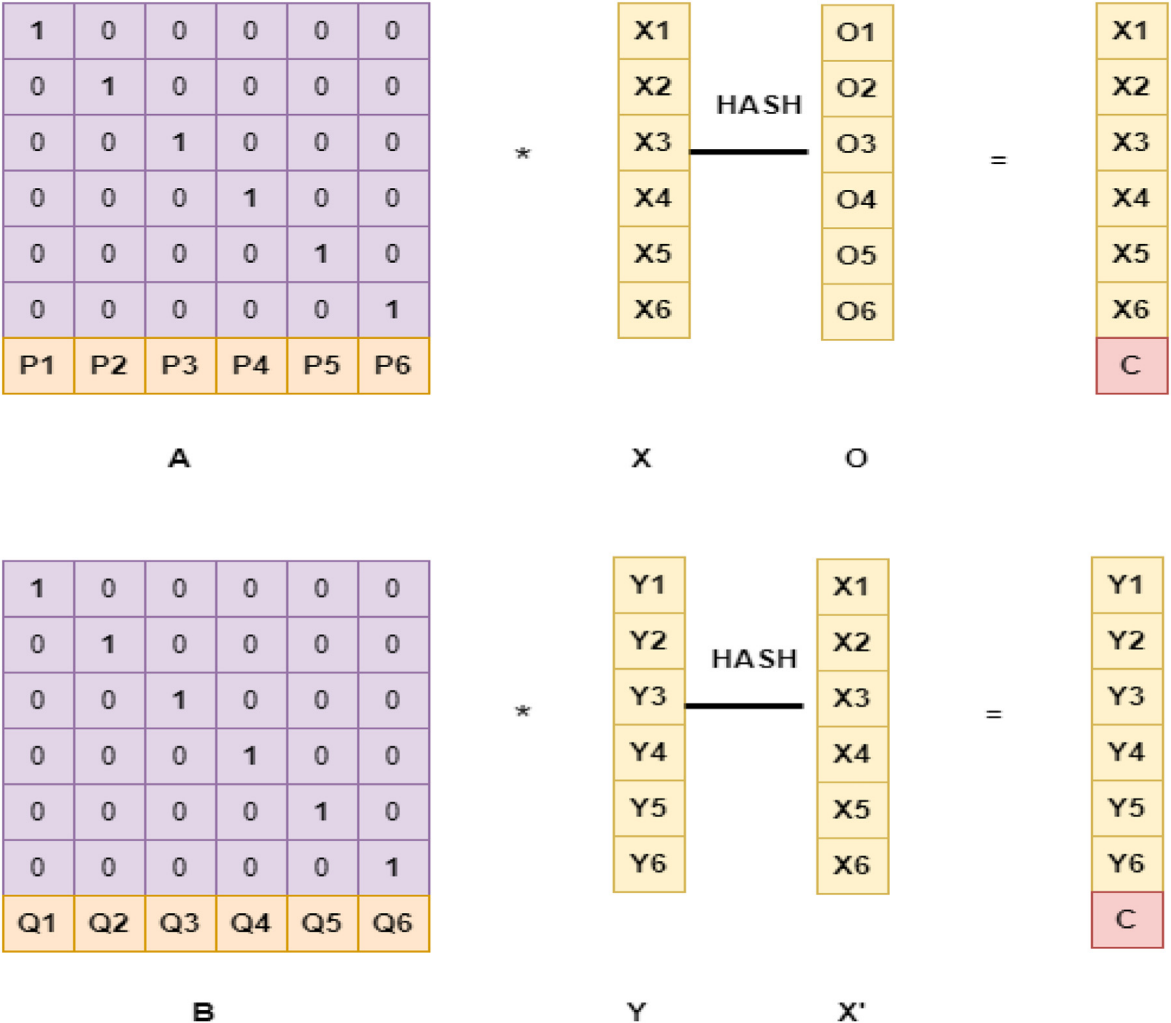
Download Process.

Fig. 3 depicts the steps an end user must take to retrieve his file from the cloud server. The initial step involves the user initiating a request, which is subsequently handled by the cloud server and subsequently consolidated among several servers. Data can be kept in several locations, such as cloud-based systems, fog computing, or local servers. Following this, the data is transmitted from the cloud server to the fog server.

By utilizing the encoding key and benefiting from the fog server's 5 % data chunks, it is possible to retrieve the entirety of the original data. In the event of this occurrence, the user is provided with the entirety of the data from the fog server. In step three, the user ultimately obtains the data from the fog server. To acquire comprehensive information, users are required to repeat the procedures outlined above.

## Experimental setup

In this study, the real-world code for implementing our system was built using the Java programming language. No simulation programme was utilized for this purpose. The Java language was utilized to implement the user registration, authentication model, and all associated coding for the fog layer and cloud layer. The data was submitted according to the specified requirements, such as in the form of text files or PDF files. However, no dataset collection was conducted for experimental analysis. The experimental purpose involved the utilization of three layers in the following manner.

### Local data storage

The data is divided into three distinct levels when using the hash-Solomon code. The first layer, called local data storage, involves storing the data on the hard drives of local devices. This approach utilizes the local device's hard disc for data storage, ensuring that data is kept close to the source for quick access.

### Fog layer storage

The split data for the fog layer was briefly saved in the MySQL databases. The data was stored in a fashion that ensured encryption. Real-time cloud computing can be employed as a Fog layer in situations when there is a substantial volume of data to be stored within the fog layer [6,12].

### Cloud layer storage

The split data for the cloud layer was encrypted and securely stored in the cloud. We utilized DriveHQ cloud services and Amazon AWS cloud services for this objective [21]. The data held on this drive was subjected to testing and evaluation using an encrypted format.

## Experimental result and discussion

The present study investigates the performance and feasibility of a three-layer server system based on fog computing, employing a novel method. The evaluation encompasses the processes of encoding, decoding, and testing using various datasets. This study demonstrates a correlation between the number of blocks and the capacity of data that an individual can store locally on their computer, employing many instances. The value denoted as M represents redundant data blocks, whereas K represents the minimum number of raw data blocks. As the value of K increases, the user's local computer's data storage capacity decreases, indicating that larger data volumes necessitate reduced device storage. The efficiency of the experiment is positively correlated with the volume of data. It is crucial to augment the value of K in the user's storage in a practical system. Consolidate the files before uploading, particularly for smaller files.

**Fig. 4:**
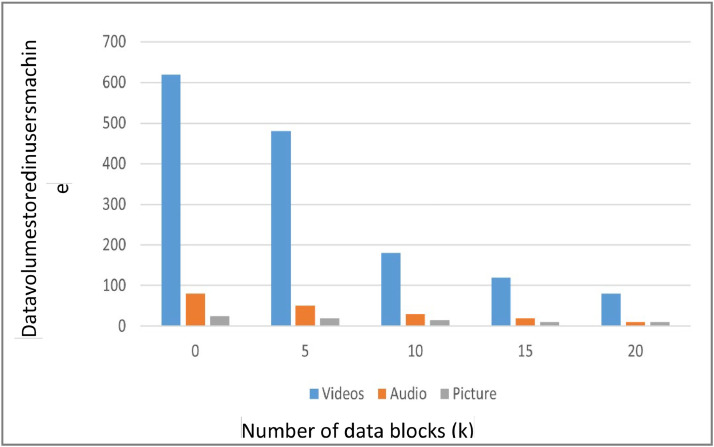
Results of Local Storage Volume of Different Types of Files.

The bar chart as shown in Fig. 4, illustrates the distribution of Videos, Audio, and Pictures over five-time intervals (0, 5, 10, 15, and 20 min). Videos start at a high count of 600 at 0 min and steadily decline to around 100 by the 20-minute mark. In contrast, Audio and Pictures remain relatively low and stable throughout the intervals, with Audio starting at about 100 and Pictures at 50, showing only slight fluctuations over time. This indicates a significant and consistent reduction in video content, while audio and picture content remain relatively unchanged.

This study contributes to the enhancement of the three-layer server structure in fog computing through an analysis of block counts, data storage capacity, and efficiency. These findings indicate that adjusting the K value to accommodate larger data volumes enhances performance and storage efficiency in real-world applications.

### Efficiency analysis

This section will explore strategies for achieving a balance between storage efficiency and coding efficiency. Storage efficiency is a crucial metric for an algorithm that deals with storage. A system that exhibits high storage efficiency can optimize storage capacity utilization. The storage efficiency is defined as:

(1) $$storageefficiency=\frac{DataSpace}{DataSpace+CheckSpace}$$

The storage efficiency in our method can be mathematically represented as Es=*k*/(*k* + *m*). Subsequently, the subsequent Eqs. (2, 3) can be derived. The storage efficiency will exhibit an upward trend as the ratio of k and m increases. It is established that an increase in the ratio of k and m leads to a corresponding rise in the number of data blocks (k), hence impacting the efficiency of coding.

(2) $$E_{s}=\frac{k}{k+m}=\;\frac{\frac{k}{m}+1}{\frac{k}{m}+1}$$

(3) $$\lim_{\frac{k}{m}\to \infty }=\frac{\frac{k}{m}}{\frac{k}{m}+1}=1$$

The efficiency of coding is directly linked to the operation performed on the Galois field [22]. We examine the impact of various coding elements that are associated with the Galois field. The Eq. (2) > *k* + *m* describes the relationship between, k, and m. As grows, there is a corresponding increase in RAM consumption. Hence, we consider the reciprocal as a representation of coding efficiency, which may be mathematically represented as:

(4) $$E_{c}=\frac{l_{n}\left(k+m\right)}{ln2}$$

**Fig. 5:**
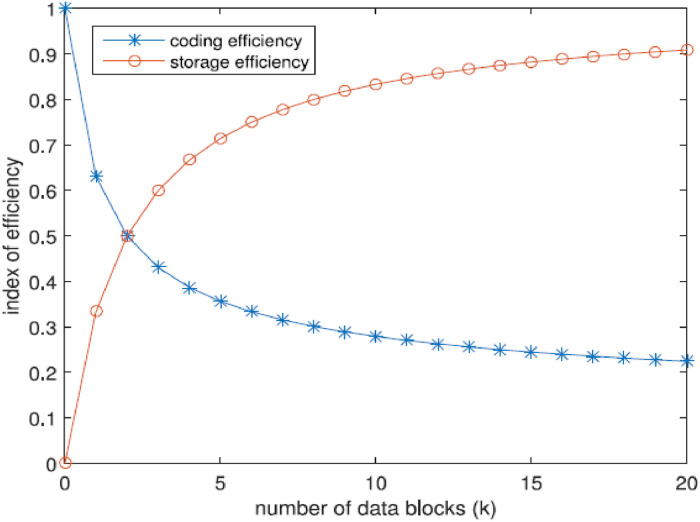
Impact of the quantity of data blocks (k) on the effectiveness of storing and coding.

Fig. 5 demonstrates the impact of increasing the number of k on both storage efficiency and coding efficiency [23]. The variable m is assigned a value of 2. The inclination towards storage efficiency contradicts the inclination towards coding. This implies that there exists a specific value of k that can attain optimal efficiency for the entire system. Hence, it is imperative to develop a novel index that incorporates both storage efficiency and coding efficiency. The overall efficiency of the plan can be mathematically represented as

The storage ratio is associated with the parameters C1 and C2. As an illustration, the variable m is assigned a value of 2, followed by the assignment of a value of 0.6 to C1 and 0.4 to C2. As depicted in Fig. 5 shows the overall efficiency of the scheme exhibits an initial increase followed by a subsequent decline upon reaching the apex of the functional graph.

(5) $$E_{\omega }=C_{1}\frac{\text{ln}\left(k+m\right)}{\ln2}+C_{2}\frac{k}{k+m}$$

**Fig. 6:**
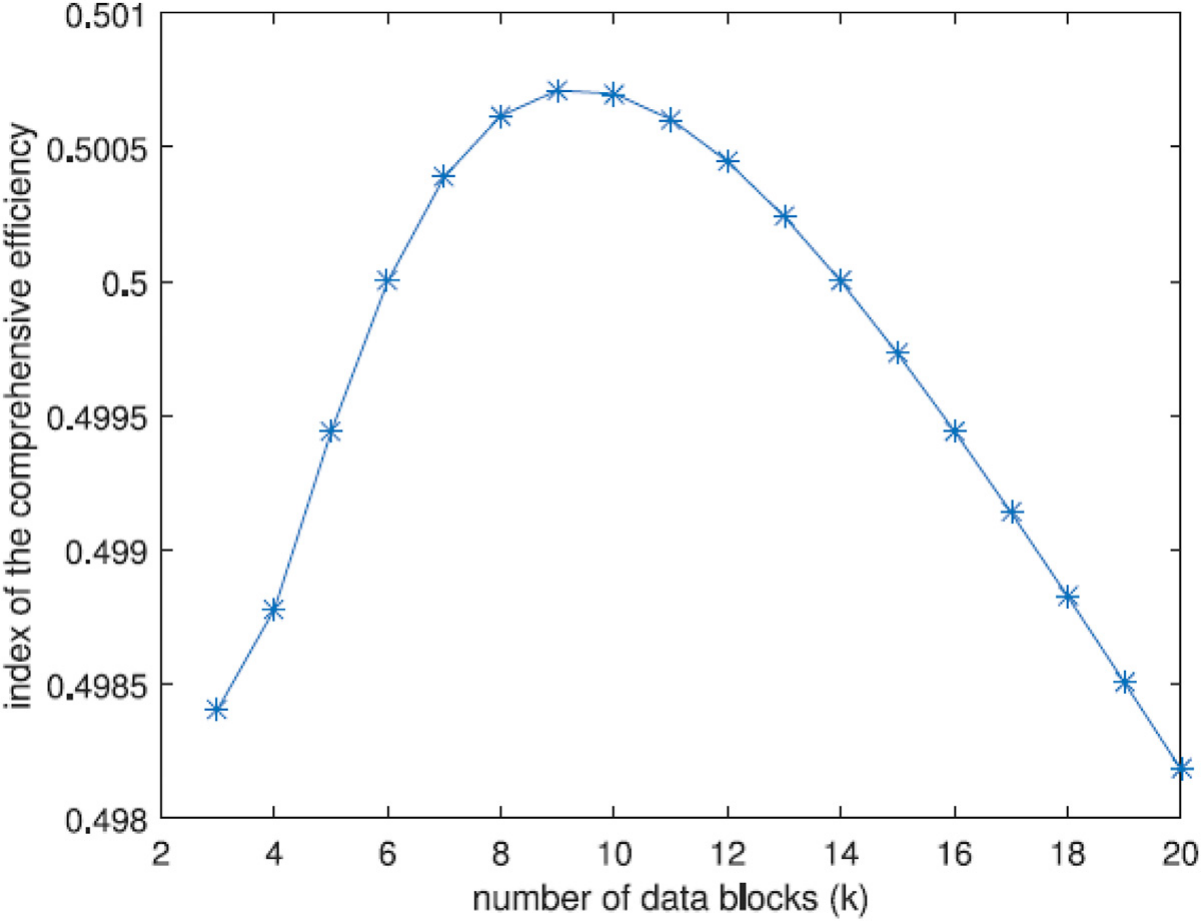
Influence of the number of data blocks (k) on the efficiency of storage and coding.

The graph drawn in Fig. 6 shows the relationship between the number of data blocks (k) and the index of comprehensive efficiency. The efficiency index starts at approximately 0.4985 for 2 data blocks, increases to a peak of about 0.5005 around 8 data blocks, and then gradually declines back to around 0.4985 by 20 data blocks. This indicates that the efficiency improves with an increasing number of data blocks up to a certain point (*k* = 8), after which it starts to decrease.

The optimal value of (k), which corresponds to the peak performance, can be regarded as the best choice for enhancing the scheme's overall efficiency. This value maximizes the effectiveness of the approach, ensuring the best possible outcomes. Identifying this ideal (k) is crucial for achieving superior performance metrics. Consequently, it is a key factor in the optimization process.

**Fig. 7:**
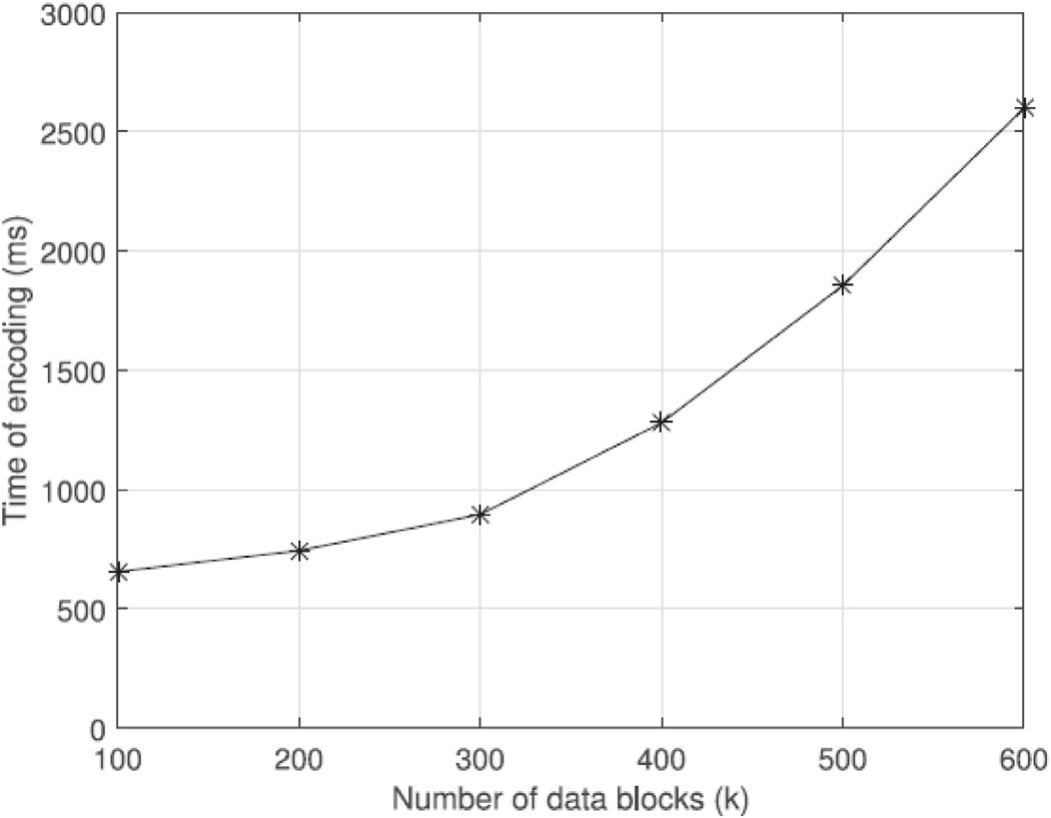
Relationship between time of decoding and the number of k.

Fig. 7 illustrates the correlation between the duration of decoding and the quantity of data blocks. Both the variable m and the variable representing the eliminated data are assigned a value of 2. When the quantity of data blocks, denoted as ‘k’, is increased from 100 to 600, there is an observed rise in the decoding time at the express speed. The decoding process requires a greater amount of time compared to the encoding process. Therefore, it is imperative to prioritize efforts aimed at improving decoding efficiency in practical situations.

Fig. 7 displays the decoding time for varying amounts of deleted data, ranging from 1 to 5. To ease storage pressure, it is advisable to maintain a low ratio between m and k. To optimize efficiency, it is advisable to download a larger amount of data as this reduces decoding time. The primary factor contributing to the efficacy of our system is the Hash-Solomon algorithm.

In Table 1, the storage metric of the fog layer and cloud layer is compared with respect to file size and encryption time. As our system splits the data in three layers, so as per the algorithm 94 % data is available on cloud layer and 4 % on Fog layer. So accordingly, when the file size of approximately 125 MB was splitted and uploaded on fog and cloud layer, the fog layer has taken 5 MB and cloud layer has taken 117 MB with encryption time as 0.128 s and 2.98 s respectively for fog layer and cloud layer.

**Table 1:** Storage metric of the fog layer and cloud layer.

	Total	Fog Layer	Cloud Layer
File Size	125MB	5MB	117MB
Encryption Time	3.2seconds	0.128 s	2.98 s

### Comparative analysis with existing work

Based on the literature analysis in section 2, the majority of research indicates that privacy in cloud storage has been enhanced through various means. Some employ diverse encryption policies in distinct locations. Some individuals address privacy concerns through the process of auditing or creating secure frameworks. Nevertheless, these investigations possess a limitation. If the CSP is not trusted, all of these measures are ineffective. The CSP has no control over internal assaults and the illicit sale of user data for personal gain. Cloud servers have the potential to be decrypted by unscrupulous attackers, irrespective of the encryption method employed [24].

In this research study, a safe cloud storage solution was introduced. Data privacy can be protected by dividing files using suitable code and implementing a TLS framework based on fog computing. This does not suggest that we abandon the use of encryption. Encryption is employed as a means of safeguarding intricate data.

The security analysis metrics have been compared to those of the existing model, with the results displayed in Table 2. This comparative analysis demonstrates the relative performance of each model. Table 2 provides a detailed comparison of the security features.

**Table 2:** Security Metric Analysis.

Metrics	Proposed Model	Existing Model
Confidentiality	High	Medium
Integrity	High	High
Availability	High	Medium
Anonymity	Very-High	Medium
Data Breach Rate	Low	Medium
Encryption Strength	AES-256	AES-128
Resilience to Attack	Low	Medium
Latency	Moderate	Low

## Conclusion

Cloud computing has enhanced the expansion of storage capacity, among other benefits. However, the utilization of online storage presents security concerns. The separation of data ownership and management arises from the fact that consumers do not have control over data storage. We present a TLS framework based on fog computing and a Hash-Solomon algorithm to guarantee privacy in cloud storage. The viability of our concept is demonstrated through a thorough theoretical safety analysis.

Optimizing the distribution of data blocks between servers can help achieve data privacy. The encoding matrix of our approach is theoretically impervious to cracking, providing additional security. Hash transformations safeguard data that is in fragmented form. The efficacy of our technique in encoding and decoding, while maintaining cloud storage economy, has been demonstrated through comprehensive experimental testing.

### Method validation

The method validation of the study involved a rigorous evaluation of the proposed three-layer server system based on fog computing. The system was tested across various datasets, focusing on encoding, decoding, and storage efficiency. The experiments demonstrated a positive correlation between the number of data blocks (K) and system performance, with optimal efficiency observed at specific values of K. Additionally, the security metrics, including confidentiality, integrity, and encryption strength, were compared against existing models, showcasing the proposed system's superiority in privacy protection. Comprehensive theoretical and experimental analyses confirmed the feasibility and effectiveness of the approach.

### Limitations

Implementing the Hash-Solomon code algorithm for data partitioning and distributing data across local machines, fog servers, and cloud storage is complex. This process requires meticulous planning and precise execution to ensure data integrity and security.

## CRediT author statement

Sneha Ojha, Priyanka Paygude: Methodology, Investigation, Validation, Writing – original draft, Visualization. Snehal Rathi, Amol Dhumane: Methodology, Supervision, Writing – review & editing. Ajay Kumar, Vijaykumar Bidve: Methodology, Writing – review & editing. Prakash Devale: Supervision, Conceptualization, Writing – review & editing.

## Ethics statements

I Confirm that this research work had not involved human subjects.

I confirm that this research work had not involved any experiments on animals:

I Confirm that this research work does not collected any data from social media platforms.

## Declaration of competing interest

The authors declare that they have no known competing financial interests or personal relationships that could have appeared to influence the work reported in this paper.

## Data Availability

No data was used for the research described in the article.
